# Laser-Assisted Diamond Cutting for Low-Damage Fabrication of High-Q CaF_2_ Whispering-Gallery Mode Resonators

**DOI:** 10.3390/mi17050581

**Published:** 2026-05-07

**Authors:** Rongbiao Yang, Tao Jia, Jiamin Rong, Huanfei Wen, Zhidong Xu, Zihan Song, Enbo Xing, Jun Tang, Jun Liu

**Affiliations:** 1 State Key Laboratory of Extreme Environment Optoelectronic Dynamic Measurement Technology and Instrument, North University of China, Taiyuan 030051, China; yangrongbiao219@163.com (R.Y.); wenhuanfei@nuc.edu.cn (H.W.); liuj@nuc.edu.cn (J.L.); 2 School of Semiconductors and Physics, North University of China, Taiyuan 030051, China; jiataonuc@163.com (T.J.); rongjiamin@126.com (J.R.); 2319014148@st.nuc.edu.cn (Z.X.); 13315994100@163.com (Z.S.); juntang@nuc.edu.cn (J.T.)

**Keywords:** ultra-precision machining, laser-assisted turning, CaF_2_ resonator, molecular dynamics

## Abstract

Calcium fluoride (CaF_2_) crystals are an ideal material for fabricating high-quality whispering-gallery-mode (WGM) optical resonators. However, their hard and brittle nature make it difficult to achieve low-damage, ultra-smooth surfaces through conventional cutting, which limits the optical performance of the resonators. To address this, laser-assisted diamond cutting technology is proposed in this study for low-damage and high-quality fabrication. Molecular dynamics simulations reveal the atomic-scale mechanism by which laser thermal effects reduce cutting forces and promote dislocation motion. Nano-scratch experiments further show that the critical depth of ductile–brittle transition (DBT) increases from 388 nm to 1070 nm, 2.76 times that of conventional cutting. Based on these results, ultra-precision turning of a high-quality hemispherical resonator with a Q factor of up to 1.3 × 10^8^ was achieved. This study provides an effective solution for low-damage and high-performance resonator fabrication from hard and brittle optical crystals.

## 1. Introduction

Calcium fluoride (CaF_2_) crystals [1,2], with their broad transparent wavelength range, stable refractive index, and excellent thermo-optical properties, have become a key material for high-end optical systems such as deep-ultraviolet lithography and high-power lasers. Particularly promising are CaF_2_ whispering-gallery mode (WGM) resonators [3,4,5] with ultra-high Q, which have shown great potential in integrated photonics, quantum information processing, and ultra-sensitive sensing [6]. These resonators operate via total internal reflection, confining optical fields within microscale volumes over extended periods, thereby significantly enhancing light–matter interaction [7,8]. Theoretically, millimeter-scale CaF_2_ WGM resonators can achieve measurement precision equivalent to kilometers of optical fiber, positioning them as an ideal platform for next-generation all-solid-state quantum sensors.

Previous studies have demonstrated that ultra-high Q factors (>10^9^) can be achieved through ultra-precision polishing techniques [9,10]. However, these approaches are typically time-consuming and lack fabrication efficiency. Single-point diamond turning combined with subsequent polishing can provide a balance between form accuracy and optical performance, but it often introduces surface and subsurface damage. Such processing-induced defects not only increase scattering loss and limit the Q factor, but also distort the intracavity field distribution and degrade mode dispersion [11,12]. Moreover, they hinder stable low-noise coupling and integration. Therefore, developing a novel manufacturing technology that offers high precision, high efficiency, and low damage has become essential to overcome the performance bottlenecks of ultra-high-Q CaF_2_ WGM resonators.

Recent advances in laser-assisted machining have demonstrated significant potential for processing hard and brittle materials, such as silicon [13], germanium [14], sapphire [15], and silicon carbide [16]. The underlying mechanism is primarily attributed to laser-induced thermal softening, which reduces cutting forces, suppresses brittle fracture, and extends the ductile machining regime [17]. For CaF_2_ crystals, several studies have investigated their deformation and material removal behavior; for example, Lee et al. [18] examined the influence of crack morphology on ductile-mode cutting performance, while Guo et al. [19] investigated the magnetoplastic scratching mechanism and the anisotropic ductile–brittle transition behavior of CaF_2_ single crystals during micro-cutting. Although substantial progress has been achieved in laser-assisted machining of various brittle materials, its application to fluoride crystals remains limited, particularly in the fabrication of WGM resonators, where ultra-smooth surfaces and minimal subsurface damage are critically required.

In this paper, we established the theoretical and experimental foundation for low-damage, high-precision machining of CaF_2_ crystal WGM resonators. By integrating a laser heat source with precision mechanical cutting, the localized mechanical properties of the material were modulated via thermal effects, enabling material removal dominated by the plastic regime and thereby fundamentally suppressing surface damage. A laser-assisted diamond cutting system was constructed and employed to fabricate a WGM optical resonator with a quality factor of 1.3 × 10^8^. Therefore, this technology is expected to provide a practical and feasible solution to the manufacturing challenges of fluoride WGM optical microcavities and further promote their applications in integrated photonics and high-sensitivity sensing.

## 2. Molecular Dynamics Simulation

**Figure 1:**
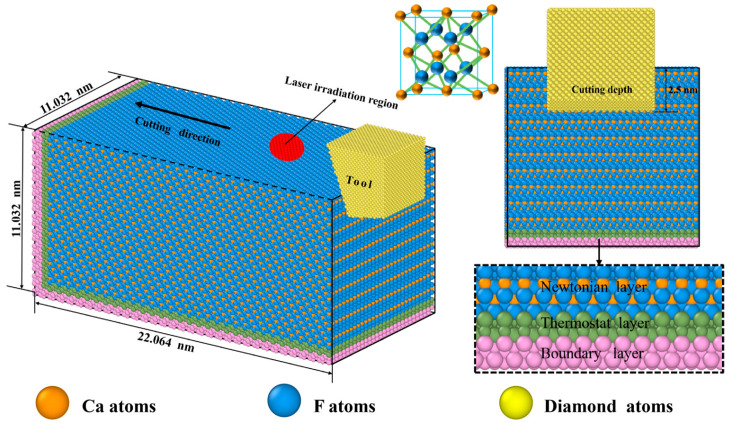
Schematic diagram of the MD model for laser-assisted cutting of CaF_2_.

In the MD simulation [20,21,22] of laser-assisted nano-cutting [23], the computational model consists of a diamond tool and a CaF_2_ crystal workpiece, as illustrated in Figure 1. For the single-point diamond turning (SPDT) machining [24,25,26] of brittle materials, employing an appropriate negative rake angle [27] helps reduce cutting force fluctuations, thereby facilitating higher machining surface quality within the plastic deformation regime. Accordingly, this model sets the geometric parameters of the diamond tool as follows: rake angle of −20°, clearance angle of 10°, and edge radius of 1 nm [28]. The tool consists of 28,117 carbon atoms and is treated as a rigid body in the simulation to eliminate interference from tool wear on the results [17,29,30].

Due to the time constraints imposed by available computational resources, reduced model dimensions and higher-than-realistic cutting velocities are typically employed, which are common characteristics of the MD approach. To mitigate potential scale effects, all geometric parameters of both the workpiece and the diamond tool were scaled proportionally using a consistent scaling factor. The workpiece is a cubic crystal system CaF_2_ crystal, whose unit cell structure is shown in Figure 1. The simulated model of the workpiece comprises a total of 177,408 atoms, with dimensions of 22.064 nm (length) × 11.032 nm (thickness) × 11.032 nm (width). Along the thickness direction, the workpiece is partitioned into three zones [31]: the boundary layer, the thermostatic layer, and the Newtonian layer. The thicknesses of both the boundary and thermostatic layers are set to 5 Å.

The interactions between ions in the CaF_2_ crystal are described using the Buckingham potential combined with long-range Coulomb interactions. The potential function is expressed as follows [19]:

(1) $$E_{B}=Ae^{-r/\rho }-\frac{C}{r^{6}},r<r_{c},$$

where parameter *A* characterizes the strength of short-range repulsive interaction between ion pairs, *ρ* is the length coefficient associated with the ion pairs, *r* is the distance between two ions, *r_c_* is the cutoff radius of the potential function, and *C* is the dispersion interaction coefficient.

The van der Waals interactions between the diamond tool and the CaF_2_ workpiece (i.e., for Ca-C and F-C pairs) are described by the Lennard-Jones (LJ) potential function [10]:

(2) $$E_{LJ}=4\varepsilon [{\left(\frac{\sigma }{r}\right)}^{12}-{\left(\frac{\sigma }{r}\right)}^{6}],$$

where *ε* represents the depth of the interaction potential well, *σ* denotes the interatomic distance at which the interaction potential is zero, and *r* is the instantaneous distance between tool and workpiece atoms. All specific parameters of the aforementioned interatomic interaction potentials used in this simulation are summarized in Table 1.

The primary cleavage plane of CaF_2_ crystal is the (111) crystallographic plane [32], along which the material is highly susceptible to cleavage fracture during machining. Given this characteristic, the (111) [$0\bar{1}1$] crystallographic direction was selected as the cutting direction in this study [33,34]. After establishing the initial configuration, the system first underwent energy minimization using the conjugate gradient method. Subsequently, a 100 ps relaxation was performed under the isothermal-isobaric ensemble to further eliminate residual stresses and bring the system to a thermodynamic equilibrium state.

In the process of simulating laser irradiation, only the thermal effect of the laser is considered, as non-thermal effects (photonic pressure) are negligible under our continuous-wave laser conditions. The laser heat source was modeled as a cylindrical moving source with a radius of 3 nm and a height of 3 nm. This dimension represents a localized effective heating zone within the computationally limited MD domain and does not directly replicate the experimental laser spot size (≈40 µm); such a scaled-down heat source is commonly adopted in MD studies of laser-assisted machining to capture local thermal effects. During the simulation of laser-assisted cutting, the laser heating zone and the diamond tool moved at the same velocity along the [$0\bar{1}1$] direction, with a depth of cut of 2.5 nm over a total cutting distance of 16 nm. By varying the heat flux (energy input per unit time) within the range of 0 to 15 eV/ps, this study evaluated the influence of thermo-mechanical coupling on the material removal mechanism of CaF_2_, with a particular focus on investigating the enhancement of the critical depth of cut for ductile-regime machining of the crystal.

**Figure 2:**
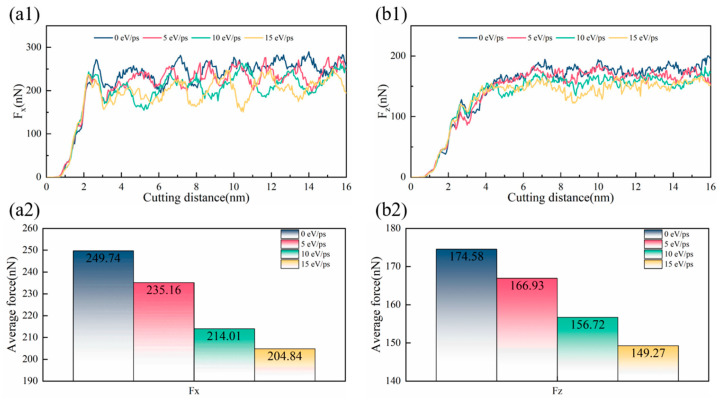
Cutting forces during laser-assisted cutting of CaF_2_ crystal. (**a1**) Evolution of tangential force (Fx); (**a2**) Statistical mean of tangential force (Fx); (**b1**) Evolution of normal force (Fz); (**b2**) Statistical mean of normal force (Fz).

Figure 2(a1,b1) illustrate the evolution of the tangential force F_X_ and normal force F_Z_ with cutting distance under different laser heat fluxes. The cutting forces decrease with increasing laser heat flux, which can be attributed to the temperature rise in the workpiece induced by laser irradiation. This thermal effect intensifies the thermal vibration of Ca^2+^ and F^−^ ions, thereby reducing both the bonding strength between ions and the stability of the crystal structure, ultimately resulting in material softening. Moreover, due to the influence of the negative rake angle, the normal force F_Z_ remains smaller than the tangential force F_X_.

The average values of F_X_ and F_Z_ during the stable cutting phase (from 4 nm to 16 nm) are summarized in Figure 2(a2,b2), respectively, for conventional cutting and laser heat fluxes of 5 eV/ps, 10 eV/ps, and 15 eV/ps. Compared to the non-laser condition, applying a laser heat flux of 5 eV/ps reduces F_X_ and F_Z_ by 14.3% and 10.2%, respectively. However, further increasing the laser heat flux to 10 eV/ps and 15 eV/ps results in only marginal additional reductions of approximately 4.3% in F_X_ and 4.7% in F_Z_. These findings indicate that localized dynamic laser heating effectively reduces cutting forces, while excessively high heat fluxes yield diminishing returns in force reduction.

**Figure 3:**
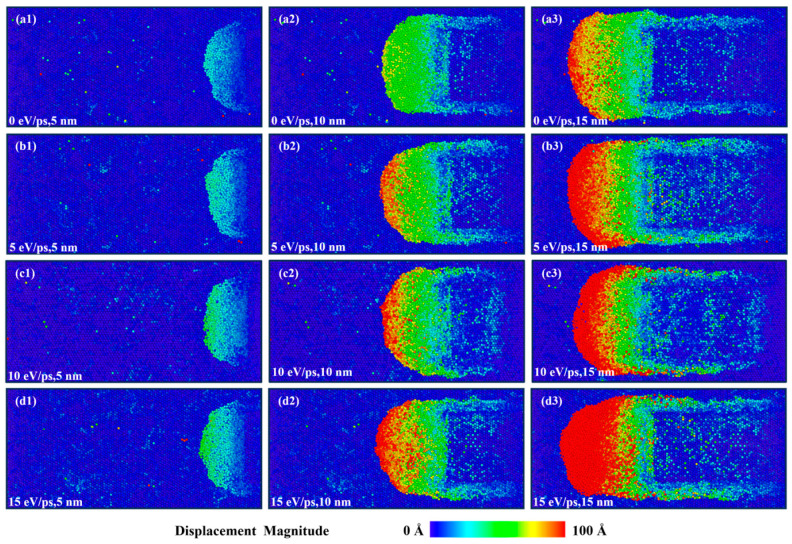
Atomic displacement under laser heating. (**a1**–**a3**): Conventional cutting; (**b1**–**b3**): 5 eV/ps; (**c1**–**c3**): 10 eV/ps; (**d1**–**d3**): 15 eV/ps.

Figure 3 presents top views of the CaF_2_ workpiece model at cutting distances of 5 nm, 10 nm, and 15 nm under different laser heat fluxes. To clearly visualize the workpiece morphology, the diamond tool is hidden, and all atoms are colored according to their displacement magnitude to facilitate the analysis of the machined surface topography. The atomic displacement on the workpiece surface primarily stems from two contributing factors: lattice vibrations induced by the laser energy input, and chips accumulated on the tool’s rake face and both sides. Comparative analysis reveals that the range and magnitude of atomic displacement during laser-assisted cutting are greater than those in conventional cutting, attributable to the enhanced atomic thermal motion excited by laser energy input. Furthermore, a comparison of the workpiece surface morphologies under different laser heat flux levels indicates that an excessively high laser heat flux leads to substantial chip accumulation on the tool’s rake face, thereby severely deteriorating the surface quality of the workpiece.

**Figure 4:**
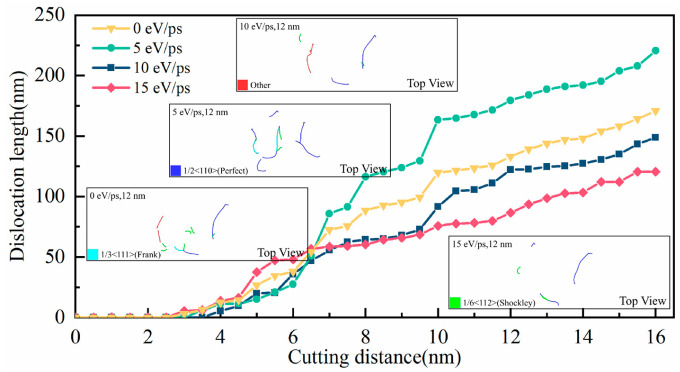
The variation in dislocation line length with time under different laser heat fluxes. The inset shows a snapshot of the dislocation distribution at a cutting length of 12 nm.

Figure 4 illustrates the evolution of dislocation length with cutting distance during the nanoscale cutting of CaF_2_ crystal under different laser heat fluxes. Overall, the dislocation length exhibits a monotonically increasing trend as cutting distance increases, confirming the continuous accumulation of plastic deformation as the tool advances. The inset in Figure 4 shows the instantaneous dislocation distribution at a cutting length of 12 nm, where dislocation lines are observed to propagate along different slip systems.

When the laser heat flux is below 5 eV/ps, the total dislocation length generated in laser-assisted cutting exceeds that in conventional cutting, indicating that moderate laser irradiation promotes dislocation nucleation and multiplication. At the microscopic level, the thermal energy from the laser reduces ionic bond strength and enhances atomic vibrational energy.

However, when the laser heat flux increases to above 5 eV/ps, the total dislocation length in laser-assisted cutting becomes slightly lower than that in conventional cutting. This shift suggests that excessive energy input raises the local temperature in the cutting zone beyond the amorphization transition threshold of the CaF_2_ crystal, leading to the collapse of the crystal structure and the formation of a widespread amorphous phase. This effectively lowers the material’s equivalent yield stress, making plastic deformation more likely to occur and macroscopically manifesting as an expanded plastic zone—the so-called “thermal softening” effect [18,35,36].

## 3. Experiments and Results

In order to validate the results of molecular dynamics simulations and quantitatively evaluate the actual improvement in the machinability of calcium fluoride (CaF_2_) crystals, laser-assisted nano-scratch experiments were conducted [37]. A single-side polished CaF_2_(111) wafer with a diameter of 20 mm and a thickness of 3 mm was used as the sample [38].

**Figure 5:**
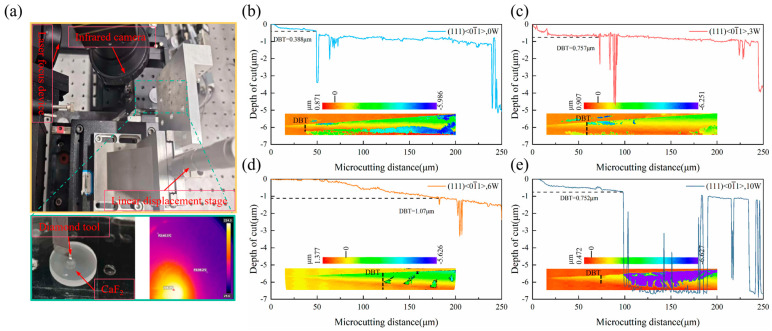
(**a**) Laser-assisted nano-scratch device (**b**–**e**) Depth of the ductile–brittle transition (DBT) under different laser powers.

The experiments were performed on a self-built laser-assisted nano-scratch system, as shown in Figure 5a. The system integrates a continuous-wave laser (CNI, maximum output power: 20 W) with a wavelength of 1064 nm and a beam spot diameter of approximately 98 μm, enabling rapid and controllable localized heating of the sample surface. A high-precision infrared thermal camera is employed for real-time monitoring and recording of the temperature field in the cutting zone. Infrared thermal imaging was employed to monitor the temperature field during laser-assisted nano-scratch experiments under different laser power conditions. The peak temperatures at the heated zone are approximately 224 °C, 315 °C, and 729 °C for laser powers of 3 W, 6 W, and 10 W, respectively. A diamond tool with a nose radius of 0.5 mm, a rake angle of −20°, and a clearance angle of 10° was used. The tool was inclined at an angle of 0.28° and advanced along the sample’s (111) [$0\bar{1}1$] crystallographic direction at a feed rate of 50 mm/min. Laser power served as the sole controlled variable in this experiment, which aimed to directly verify the thermal effect predicted by the simulations. To evaluate repeatability, three independent nano-scratch experiments were performed under each laser power condition. After scratching, the morphology of the scratches was characterized in three dimensions using an Olympus LEXT OLS5000 laser confocal microscope (Olympus Corporation, Tokyo, Japan). The critical depth of the ductile–brittle transition (DBT) was determined for each test, and the results show good consistency across measurements.

Figure 5b–e reveals the influence of laser power on the DBT [39,40] of CaF_2_ crystals, which aligns closely with the simulation predictions. As the laser power increased from 0 W, the critical depth for the transition exhibited a non-monotonic trend, first increasing and then decreasing [41,42,43]. Without laser assistance, the critical depth was only 388 nm, with evident micro-cracks and material chipping at the scratch edges, displaying typical brittle fracture characteristics. Introducing a 3 W laser increased the depth to 757 nm. When the power was raised to 6 W, the critical depth reached 1070 nm, 2.76 times that of conventional cutting. The scratch edges at this depth were smooth and continuous, with no visible cracks, presenting a typical plastic flow morphology.

However, when the laser power was further increased to 10 W, although the initial critical depth remained superior to that of conventional cutting, severe material re-deposition and surface oxidation appeared at the end of the scratch, and the critical depth decreased to 752 nm. Topographical analysis indicated that the scratch surface under high laser power exhibited uneven melting-solidification features. This observation is consistent with the “excessive amorphization inhibiting dislocation motion” mechanism predicted by the simulations. The phenomenon suggests that while excessive thermal input can sustain some plastic removal, it does so at the cost of surface quality and introduces subsurface thermal damage, thereby adversely affecting the final machining outcome.

**Figure 6:**
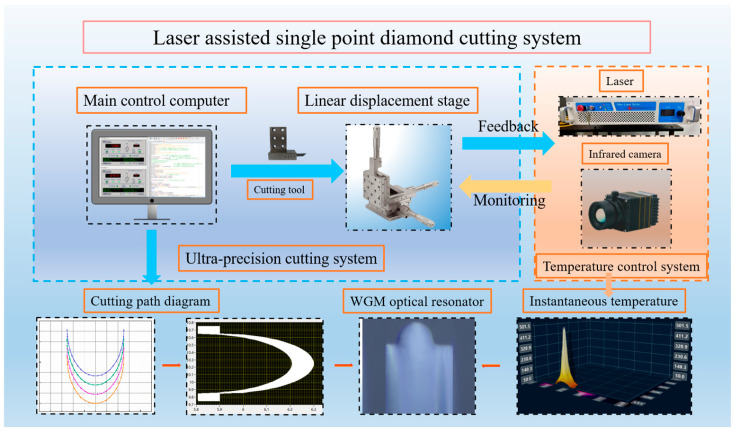
Laser-assisted cutting system for WGM resonator fabrication.

Based on the determination of the optimal laser power process for laser-assisted cutting, we designed and constructed a dedicated laser-assisted nanoscale cutting system (Figure 6) to achieve high-precision thermo-mechanical coupled machining. It organically integrates key technologies such as ultra-precision motion control, localized heat source management, real-time process monitoring, and closed-loop feedback. The core consists of two synergistically operating subsystems: the master control and thermo-mechanical coupling management subsystem, as well as the ultra-precision cutting and forming subsystem.

The master control and thermo-mechanical coupling management subsystem coordinates the entire system with the master control computer as its central hub. On one hand, it controls the linear displacement stage to execute pre-programmed cutting paths with nanometer-level accuracy; on the other hand, it synchronously drives the laser to output energy, ensuring the laser beam is precisely focused onto a micro-area of the workpiece material ahead of the diamond tool’s cutting edge to achieve localized heating. The subsystem integrates an infrared thermal imager to monitor the temperature field distribution in the cutting zone in real time. The captured data is fed back to the control system, where a dedicated algorithm dynamically adjusts the laser power to maintain the processing temperature within the optimal range, thereby achieving precise and stable control of the “thermal softening” effect.

The ultra-precision cutting and forming subsystem, under the unified control of the computer, drives the diamond tool to move along a complex three-dimensional cutting path. The core function of this subsystem, as represented by the schematic of the cutting coil trajectory in Figure 6, is its ability to directly machine complex curved microcavity structures with smooth sidewalls on CaF_2_ wafers through precise tool path planning and motion control. The main turning parameters for hemispherical resonator fabrication were as follows: spindle speed of 500 rpm, feed rate of 0.1 mm/min, depth of cut per pass of 1 μm, and no coolant used. A diamond tool with a nose radius of 0.05 mm, a rake angle of 0°, an included angle of 18°, and a clearance angle of 8° was selected. This allows for the one-step formation of the main body of a WGM resonator, replacing the traditional multi-step and time-consuming grinding process.

**Figure 7:**
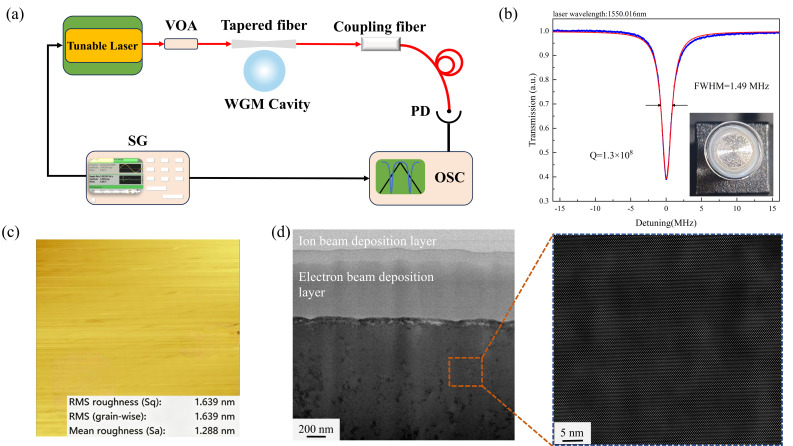
(**a**) Schematic of the measurement system, including a tunable laser (Toptica DLCpro, Toptica Photonics AG, Gröfelfing, Germany), variable optical attenuator (VOA), photodetector (PD, Thorlabs B835C, Thorlabs Inc., Newton, NJ, USA), and oscilloscope (OSC, Tektronix MSO64, Tektronix Inc., Beaverton, OR, USA), signal generatoretc (SG, Tektronix 31000, Tektronix Inc., Beaverton, OR, USA), etc. (**b**) Optical performance test of the high-precision hemispherical WGM optical resonator. (**c**) AFM surface roughness measurement. (**d**) Subsurface TEM image of the CaF_2_.

Using this integrated system, a high-precision hemispherical WGM optical resonator was fabricated by turning an 8 mm diameter, 1 mm thick CaF_2_ wafer under a laser power of 6 W. After ultrasonic cleaning, the resonator was directly characterized in the as-turned state without any post-machining mechanical polishing. The waveguide region of the resonator was then scanned with high resolution using an atomic force microscope (AFM). To ensure the representativeness of the measurements, four typical regions were selected on each sample for repeated scanning, including one central region and three edge regions. The scan area was set to 10 μm × 10 μm to capture both surface texture and local topographical variations. The AFM results (Figure 7a) show that the measured arithmetic average roughness (Ra) is 1.28 nm and the root-mean-square roughness (Rq) is 1.64 nm. These observations directly indicate that laser-induced localized thermal softening shifts the material removal mechanism toward plastic flow, thereby enabling the generation of a near-atomic-level flat surface.

As shown in Figure 7c, the subsurface region of the CaF_2_ crystal cavity after laser-assisted cutting exhibits a continuous, dense, and structurally uniform lamellar feature, with an overall homogeneous contrast distribution and no obvious accumulation of nanoparticles or pore structures. High-resolution TEM results further indicate that no significant grain refinement is observed in this region; instead, the crystal structure remains relatively intact or evolves into a uniform metastable structural layer. This structural state reduces refractive-index fluctuations within the material. The formation of this dense layer may be associated with localized thermal effects or structural relaxation during machining. Under laser thermal irradiation, the near-surface material undergoes local rearrangement and even short-range reconstruction, which partially repairs the pre-existing damage structure and thereby leads to the formation of a dense and uniform surface layer. However, owing to the limited depth of energy penetration, such structural optimization is mainly confined to the extreme surface region.

Subsequently, we conducted optical performance tests on the fabricated WGM resonator. The quality (Q) factor was measured based on the full width at half maximum (FWHM) method using a tapered fiber coupling system. A narrow-linewidth laser with a central wavelength of 1550 nm was employed, modulated by a triangular wave at 3 V and 10 Hz for scanning. After optimizing the coupling condition, the transmission spectrum was recorded, and the temporal FWHM was obtained through Lorentzian fitting. The optical quality factor of the microcavity was calculated as 1.3 × 10^8^ (Figure 7b).

## 4. Conclusions

This study demonstrates that laser-assisted diamond cutting transitions the material removal mechanism in CaF_2_ from brittle fracture to plastic flow by thermally promoting dislocation activity, as confirmed by simulations and experiments. An optimal process was established using a self-built system, increasing the brittle-to-ductile transition critical depth from 388 nm to 1070 nm, representing a 2.76-fold improvement. Consequently, an integrated hemispherical optical resonator with a surface roughness of Ra = 1.28 nm and an optical quality factor of 1.3 × 10^8^ was fabricated.

This work deepens the understanding of laser–material interactions in hard-brittle materials and provides a practical solution for the precision machining of hard-brittle optical crystals. It holds importance for promoting the development of integrated photonics and quantum information technologies. Future work will further extend the applicability of this technology to different hard-brittle optical materials and complex micro-nano structures, while exploring its integration with subsequent processes such as annealing to continuously approach the theoretical performance limits of the devices.

## Figures and Tables

**Table 1:** Parameters of the interatomic potentials used in the study.

Atom Pair	Potential Style	Parameters	Values
Ca-F	Buckingham	*A* (eV)	1272.8
		*ρ* (Å)	0.2997
		*C* (eV/Å^6^)	0
F-F	Buckingham	*A* (eV)	99,731,833.99084
		*ρ* (Å)	0.12013
		*C* (eV/Å^6^)	17.02423
Ca-C	Lennard-Jones	*ε* (eV)	0.003
		*σ* (Å)	3.69
F-C	Lennard-Jones	*ε* (eV)	0.0036
		*σ* (Å)	3.69

## Data Availability

The experimental data that support the findings of this study, including nano-scratch test results, surface roughness measurements, and quality factor (Q-factor) values, are available from the corresponding author upon reasonable request.
